# The *ABCC4* gene is associated with pyometra in golden retriever dogs

**DOI:** 10.1038/s41598-021-95936-1

**Published:** 2021-08-17

**Authors:** Maja Arendt, Aime Ambrosen, Tove Fall, Marcin Kierczak, Katarina Tengvall, Jennifer R. S. Meadows, Åsa Karlsson, Anne-Sofie Lagerstedt, Tomas Bergström, Göran Andersson, Kerstin Lindblad-Toh, Ragnvi Hagman

**Affiliations:** 1 Faculty of Health and Medical Sciences, Department of Veterinary Clinical Sciences, University of Copenhagen, Copenhagen, Denmark; 2 Science for Life Laboratory, Department of Medical Biochemistry and Microbiology, Uppsala University, Uppsala, Sweden; 3 Department of Clinical Sciences, Swedish University of Agricultural Sciences, Uppsala, Sweden; 4 Department of Medical Sciences, Molecular Epidemiology and Science for Life Laboratory, Uppsala University, Uppsala, Sweden; 5 Department of Cell and Molecular Biology, National Bioinformatics Infrastructure Sweden, Science for Life Laboratory, Uppsala University, Uppsala, Sweden; 6 Broad Institute of MIT and Harvard, Cambridge, MA USA; 7 Department of Animal Breeding and Genetics, Swedish University of Agricultural Sciences, Uppsala, Sweden

**Keywords:** Genome-wide association studies, Animal disease models, Genetic models

## Abstract

Pyometra is one of the most common diseases in female dogs, presenting as purulent inflammation and bacterial infection of the uterus. On average 20% of intact female dogs are affected before 10 years of age, a proportion that varies greatly between breeds (3–66%). The clear breed predisposition suggests that genetic risk factors are involved in disease development. To identify genetic risk factors associated with the disease, we performed a genome-wide association study (GWAS) in golden retrievers, a breed with increased risk of developing pyometra (risk ratio: 3.3). We applied a mixed model approach comparing 98 cases, and 96 healthy controls and identified an associated locus on chromosome 22 (*p* = 1.2 × 10^–6^, passing Bonferroni corrected significance). This locus contained five significantly associated SNPs positioned within introns of the *ATP-binding cassette transporter 4* (*ABCC4*) gene. This gene encodes a transmembrane transporter that is important for prostaglandin transport. Next generation sequencing and genotyping of cases and controls subsequently identified four missense SNPs within the *ABCC4* gene. One missense SNP at chr22:45,893,198 (p.Met787Val) showed complete linkage disequilibrium with the associated GWAS SNPs suggesting a potential role in disease development. Another locus on chromosome 18 overlapping the *TESMIN* gene, is also potentially implicated in the development of the disease.

## Introduction

Purulent bacterial infection of the uterus (pyometra) is one of the most common diseases of intact female dogs. On average one in five female dogs are diagnosed with the disease before 10 years of age^1,2^. The proportion of affected bitches diagnosed varies greatly between different breeds, i.e. some breeds develop the disease to a much larger extent and at an earlier age than others (from 3% in Finnish spitz’ to 66% in Bernese mountain dogs)^1,2^. The clear breed predisposition indicates that genetic risk factors play a role in the pathogenesis. The golden retriever breed is among the breeds that have increased risk of pyometra (age corrected risk ratio 3.3)^2^. By 10 years of age, approximately 37% of all intact Swedish female golden retrievers will have been affected by the disease^1,2^.

Pyometra is a potentially life-threatening illness that develops as a consequence of a combination of hormonal and bacterial factors. During the luteal phase of the oestrus cycle, high progesterone hormone levels make the uterus susceptible to opportunistic bacterial infections, foremost by *Escherichia coli*. Infection of the uterus can lead to sepsis and related endotoxemia and organ dysfunctions in severely affected individuals. In addition, circulating inflammatory mediators increase^3,4^. The treatment of choice is surgical ovariohysterectomy. Non-surgical treatment alternatives are possible in less severe cases, but are frequently associated with disease relapse^5^.

Diseases of the reproductive organs, such as pyometra, are more commonly diagnosed in Sweden in comparison to many other countries, where in the latter, most non-breeding female dogs are spayed for reproduction preventive purposes^6^. Of all Swedish dogs, 90% are insured and 67% are registered in the Swedish Kennel Club (SKK), which facilitates identification of cases and control dogs suitable for genetic research studies through insurance company databases and the SKK registry^7^.

Here we present an investigation of Swedish golden retrievers to identify genetic risk factors for pyometra using a genome-wide association study approach with a case–control population consisting of clinically well-defined affected and healthy dogs.

## Results

### Genome-wide significant locus on chromosome 22 in CanFam 3.1

**Figure 1:**
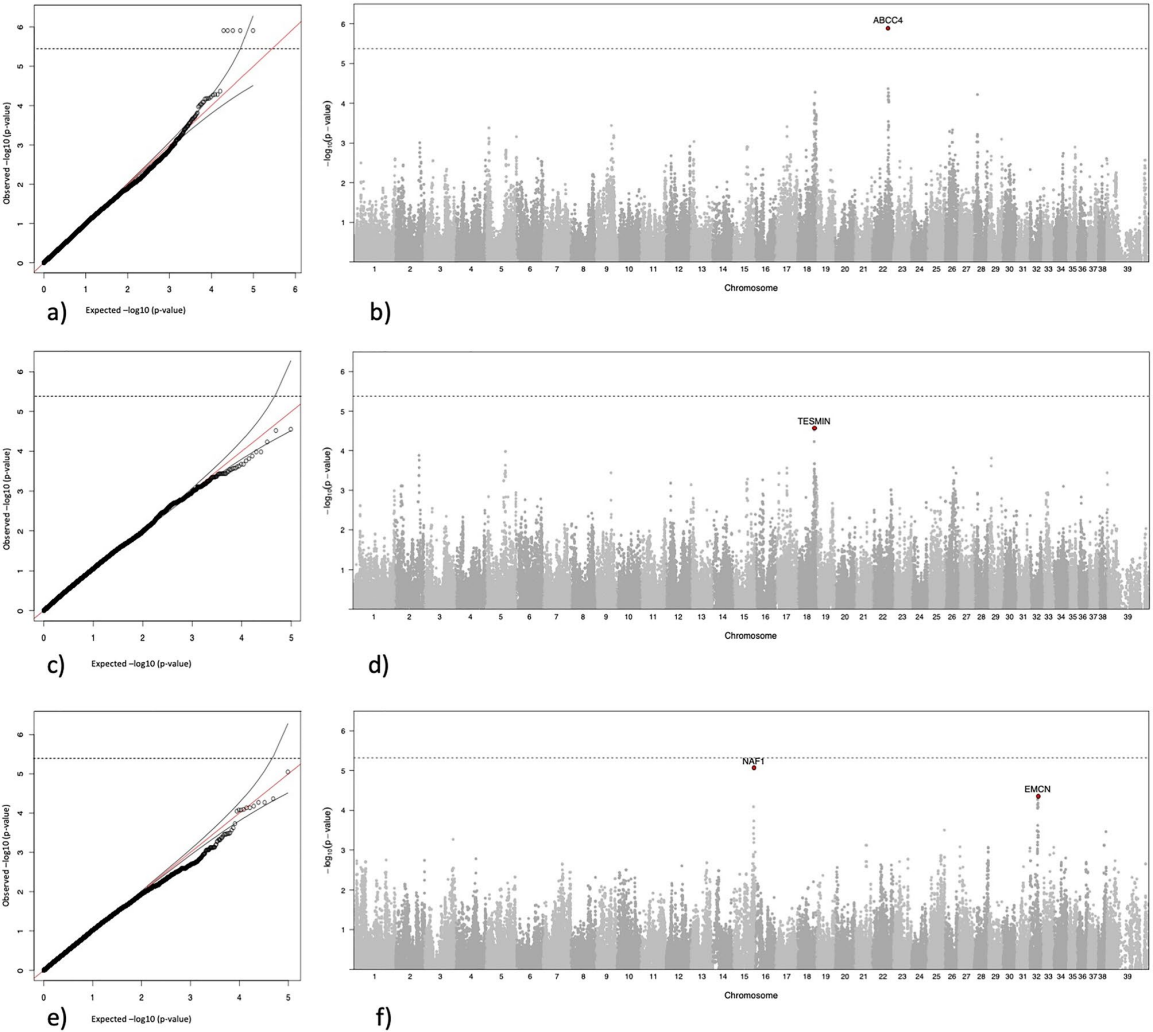
GWAS of pyometra in golden retrievers. (**a**) QQ-plot (*λ* = 0.99) and (**b**) Manhattan plot for GWAS of 98 cases and 96 controls identified a genome-wide significant signal on chromosome 22. Stippled line shows Bonferroni corrected significance threshold. (**c**) QQ-plot and (**d**) Manhattan plot of conditional GWAS using the genotype for one of the most associated SNPs (chr22: 45,875,420) on chromosome 22 as covariate, illustrating a slightly stronger association on chromosome 18, and the disappearance of the association on chromosome 28. (**e**) QQ-plot and (**f**) Manhattan plot for age of onset analysis showed two suggestive loci on chromosome 15 and 32.

To identify disease-associated loci, 194 female golden retrievers were genotyped using the 170 k CanineHD BeadChip. Ninety-eight of the dogs were classified as cases and 96 as controls. The mean age of onset for the cases was 6.6 years (SD 2.1 years). All controls were intact and > 7 years old with a mean age of 8.6 years (SD 1.4 years). At initial quality control and filtering, 1000 SNPs were removed for low genotyping rate (< 95%) and 72,878 SNPs were removed for having a minor allele frequency of less than 5% leaving 97,468 SNPs for further analysis. No individuals were removed for having a low genotyping rate and the average genotyping rate in the population was 99%. A multidimensional scaling plot was generated showing the first two dimensions (C1-C2) (Figure S1). No clustering between cases versus controls was noted in the population as a whole. Calculation of relatedness showed that two control dogs were related at sibling level (PI_HAT 0.51, both individuals were kept). A GWAS was performed using EMMAX to account for cryptic relatedness between individuals and population structure^8^. One genome-wide significant locus containing 5 SNPs in complete LD was identified on chromosome 22 at ~ 45 Mb, *p* = 1.24 × 10^–6^, which was below the LD-corrected Bonferroni significance threshold calculated as 4.2 × 10^–6^ (see QQ-plot and Manhattan plot in Fig. 1a, b). The QQ-plot did not show evidence of inflation (lambda 0.99) with the associated SNPs on chromosome 22 above the dotted line reaching Bonferroni corrected significance.

Two tentative additional loci were seen in the Manhattan plot (Fig. 1b). A locus on chromosome 18 (top SNP chr18:51,224,157, *p* = 5.2 × 10^–5^), and a locus on chromosome 28 (top SNP chr28: 8,872,257, *p* = 6.0 × 10^–5^). None of these reach Bonferroni corrected significance.

### Conditioning on the top locus

**Figure 2:**
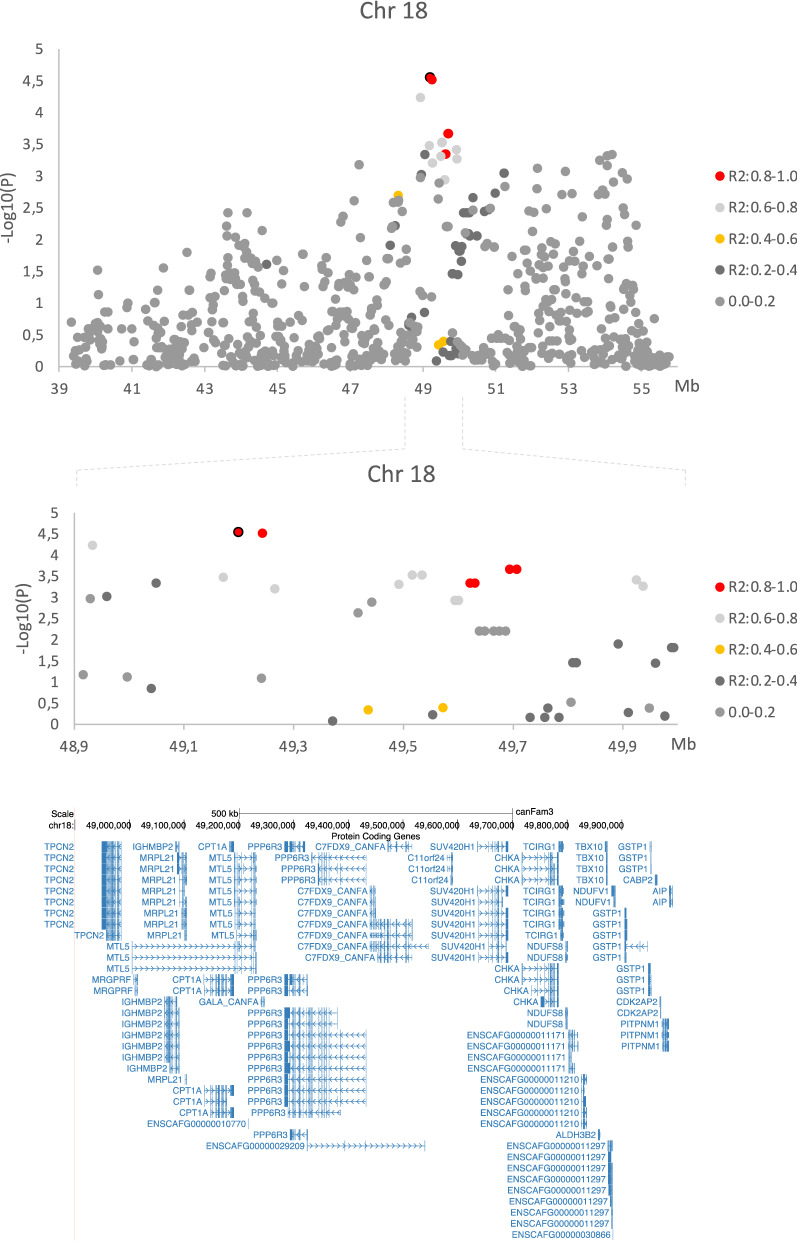
Detailed view of the associated locus on chromosome 18. (**a**) Zoomed in view of chromosome 18. The LD R^2^-value between the most highly associated SNP is illustrated by colour. (**b**) Further zoomed in view showing chr18 48.9–50 Mbp. (**c**) annotation of the region chr18 48.9–50 Mbp based on the UCSC browser annotation.

To evaluate if either of the two additional loci represented independent risk factors from the chromosome 22 locus, a conditional genome-wide analysis was performed choosing the genotype of one of the top-associated SNPs (chr22:45,875,420) on chromosome 22 as a covariate. As seen in the QQ plot (Fig. 1c) and Manhattan plot (Fig. 1d), the locus on chromosome 18 shifted ~ 2 Mb but showed a mildly improved p-value leaving it as a suggestive locus (chr18:49,198,998, *p* = 2.8 × 10^–5^), whilst the association to the SNP on chromosome 28, located in intron 7 of the *SORBS1* gene, disappeared. The most significantly associated SNP on chromosome 18 identified in this analysis was located in intron 4 of the *TESMIN (MTL5)* gene (the allele frequency was 0.64 in cases and 0.44 in controls). A closeup of the locus on chromosome 18 including the LD structure and annotation of the region can be found in Fig. 2.

The risk alleles on chromosome 22 are present in 40% of the cases, however when looking at the distribution of alleles for the chromosome 22 and chromosome 18 loci (at 49 Mb) then 96% of the cases carried at least one risk allele from one of the two loci versus only 70% of the controls. The distribution of genotypes between cases and controls is shown in Figure S2.

### Association to age of onset

To investigate potential loci associated to early onset of pyometra, we performed an association analysis within the cases only, using age of onset in days as a continuous variable. No SNPs reached Bonferroni corrected significance (Fig. 1e, f). Two loci on chromosome 15 and 32 stood out and were considered as suggestively associated. The most strongly associated SNP on chromosome 15 (chr15:59,440,763, *p* = 9.0 × 10^–6^) is located within intron 3 of the *Nuclear Assembly Factor 1 ribonucleoprotein* (*NAF1*) gene. The most strongly associated SNP on chromosome 32 (chr32:22,285,412, *p* = 4.32 × 10^–5^) was located in intron 1 of the *Endomucin* (*EMCN*) gene.

### Investigation of top locus identifies non-synonymous SNP in the *ABCC4* gene

**Figure 3:**
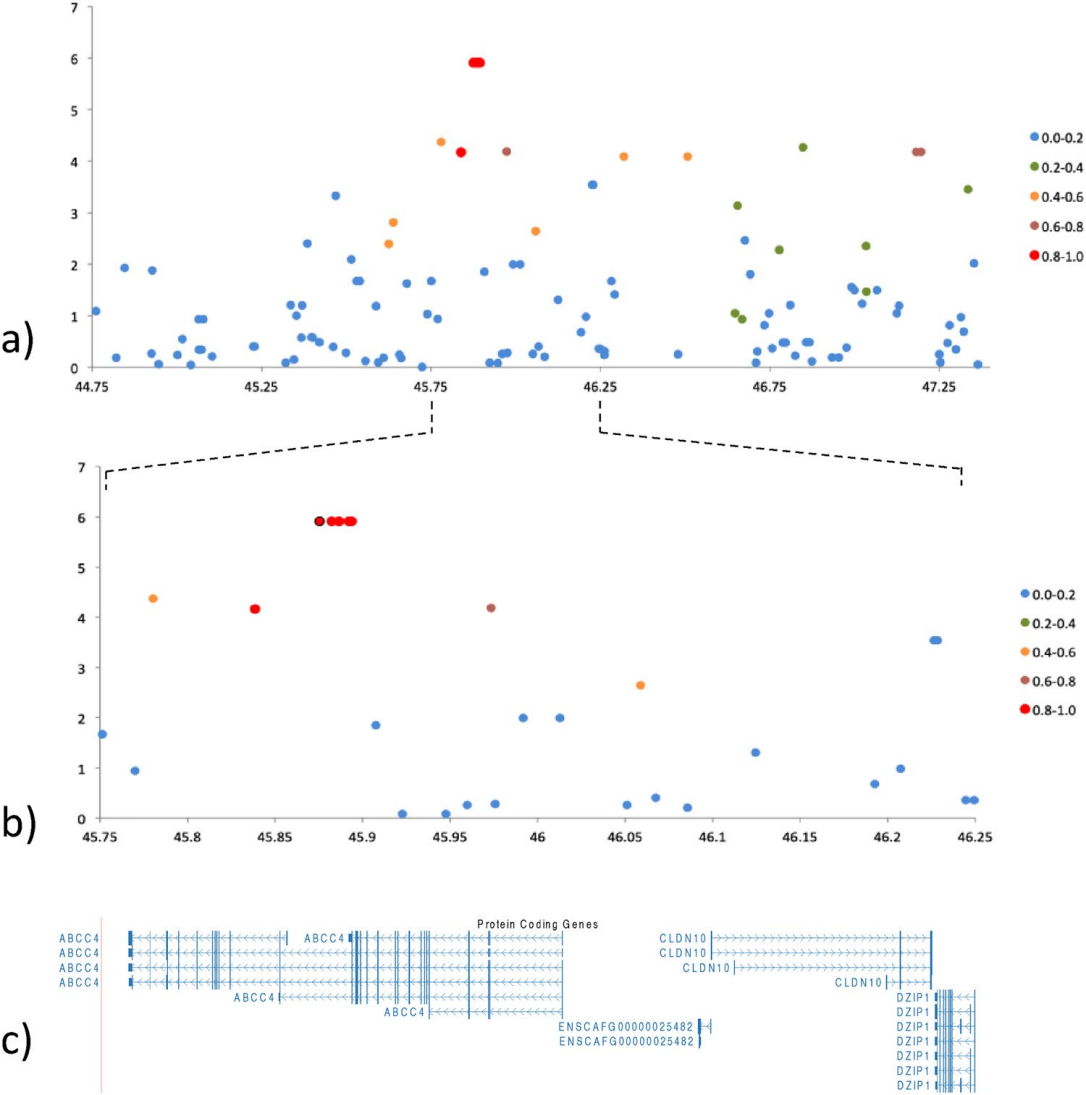
Detailed view of the associated locus on chromosome 22. (**a**) Zoomed in view of chromosome 22 at 44.7–47.2 Mbp. The LD R^2^-value between the most highly associated SNPs is illustrated by colour. (**b**) Further zoomed in view of the most associated SNPs on chromosome 22 cfa 45.7–46.2 Mbp. (**c**) The *ABCC4* gene is located within the chromosome 22 45.7–46.2 Mbp region.

The genome-wide significant locus on chromosome 22 was defined as an 18.2 kb haplotype block of 5 GWAS SNPs in complete LD (r^2^ = 1.00, chr22:45,875,420–45,893,599 bp) spanning introns 18 and 19 of the *ATP-binding cassette transporter 4* gene (*ABCC4*, ENSCAFG00000005433, ENSCAFT00000008769, UniProt F1PNA2), (Fig. 3a–c). The allele frequency for the risk haplotype was 0.21 in the cases versus 0.05 in the controls (Table 1), resulting in an odds ratio of 4.8 (95% CI 2.3–9.9). A summary of the allele frequencies and *p* values for the five GWAS SNPs is shown in Table 1.

**Table 1:** Significantly associated GWAS SNPs.

SNP Id	Chr	Bp	Allele risk/non-risk	Location in relation to *ABCC4* gene	Risk allele freq. (cases)	Risk allele freq. (controls)	GWAS *P* value
BICF2G630333328	22	45,875,420	T/G	intron 19/30	0.21	0.05	1.24e−06
BICF2G630333353	22	45,882,260	A/G	intron 19/30	0.21	0.05	1.24e−06
BICF2G630333364	22	45,886,617	T/G	intron 19/30	0.21	0.05	1.24e−06
BICF2G630333376	22	45,892,301	G/A	intron 19/30	0.21	0.05	1.24e−06
BICF2G630333380	22	45,893,599	C/T	intron 18/30	0.21	0.05	1.24e−06

The CanFam3.1 genomic coordinates are shown for the five associated GWAS SNPs reaching Bonferroni corrected significance. Allele frequency in cases and controls for the 98 cases and 96 controls is shown.

To further investigate the associated locus on chromosome 22, we generated whole genome sequencing data from a pool of 10 pyometra cases (22X mean coverage), which were all heterozygous for the associated risk haplotype on chromosome 22. In addition, sequencing data was generated from one individual homozygous for the GWAS risk haplotype (23X coverage) and 10 individually barcoded individuals homozygous for the non-risk alleles (3 cases and 7 controls; 4.4X mean coverage). A 0.25 Mb region covering the *ABCC4* gene (chr22:45,767,063–46,013,484 bp) was extracted from the sequencing data and called variants were annotated. In total 1,051 SNPs were identified in the region, out of which 627 were known variants. Four missense variants were identified within the *ABCC4* gene (Table 2), one of which, chr22:45,893,198 (rs8937218), was located within the associated GWAS locus.

**Table 2:** Fine-mapped SNPs with potential function in the chr22 region.

Chr	Position	Reference	Alternative	Risk allele	Amino acid change	SIFT score	LD with GWAS LOCUS	*P* value GWAS (98 cases-96 controls)	Allele frequency (98 cases/96 controls) risk allele	Dominant amino acid 100 mammals
chr22	45,815,581	G	A	A	972 [A/T]	Tolerated (0.53)	0.64	4.9 × 10^–4^	0.14/0.03	A (T not seen in other species)
chr22	45,823,359	G	A	G*	874 [V/I]	Tolerated (1)	Nil	Nil	1.0/1.0	I (V seen in mouse, shrew, dog )
chr22	45,893,198	A	G	A	787 [M/V]	Tolerated (1)	1.00	1.2 × 10–6	0.21/0.05	V (M seen in pig, naked mole rat, chinchilla, brush tailed rat, dog, lizard and flared flycatcher)
chr22	45,934,522	C	T	T	297 [A/V]	Tolerated (0.08)	0.01	0.23	0.74/0.69	A and S (V not seen in other species)

The four missense SNPs in the *ABCC4* gene are displayed. They were identified in the 45–46 MB region of chromosome 22 based on next generation sequencing. The reference, risk and protective allele for each SNP is listed as well as the associated p-values for the SNPs when incorporated into the GWAS data set for 98 cases and 96 controls. The r^2^ value is listed in relation to LD calculations between the most associated GWAS SNP on chromosome 22 and each of the coding SNPs. An evaluation of the most common amino acid residues for the *ABCC4* coding changes based on the UCSC genome browser is also noted. *Only one dog (control) in the extended dataset with 292 dogs was heterozygous for the chr22:45,823,359 SNP.

To expand the study of the *ABCC4* gene in a larger population of golden retrievers, we designed TaqMan genotyping assays for the four identified missense variants. Genotyping of these selected SNPs was carried out in 292 golden retrievers including 134 cases and 158 controls. The 292 dogs included the 97 cases and 96 controls, which were part of the GWAS analysis (See supplementary Figure S3 for overview of data). The additional dogs were individuals who were not chosen to be part of the GWAS analysis due to relatedness. For the dogs, which did not have 170 k genotyping data available, two of the GWAS SNPs (chr22:45,882,260 and chr18:49,198,998) were also genotyped. The TaqMan data from the original GWAS dogs was merged with the GWAS dataset.

A genome-wide association analysis was repeated on the merged GWAS dataset using a mixed model approach (EMMAX). One of the *ABCC4* coding SNPs (Chr22: 45,893,198) was in complete LD (r^2^ = 1.00) with the identified GWAS locus on chr 22: i.e. equally associated with the disease phenotype based on the *p* value (*p* = 1.24 × 10^–6^, lambda 0.99). This coding sequence variant (A > G) causes an amino-acid substitution p.Met787Val in the encoded ABCC4 protein. The SIFT score for the amino acid change is 1.0 indicating that this is likely to be a well-tolerated change.

When performing a basic association test using PLINK 1.07 for the six TaqMan SNPs for all 292 genotyped dogs, three dogs were removed for low genotyping rate < 0.5. For the remaining dogs, complete LD with a r^2^ = 1.00 was seen between two SNPs chr22: 45,882,260 and chr22:45,893,198 indicating that the risk haplotype seen in the smaller GWAS dataset is still present in this larger population. For this basic association including all TaqMan genotyped individuals the best association was to the chr18:49,198,998 SNP with a *p* value of 7.89 × 10^–5^ with the candidate SNP chr22:45,893,198 being less associated *p* = 1.48 × 10^–4^ and with a mildly reduced OR 2.6 (95% CI 1.6–4.5).

**Figure 4:**
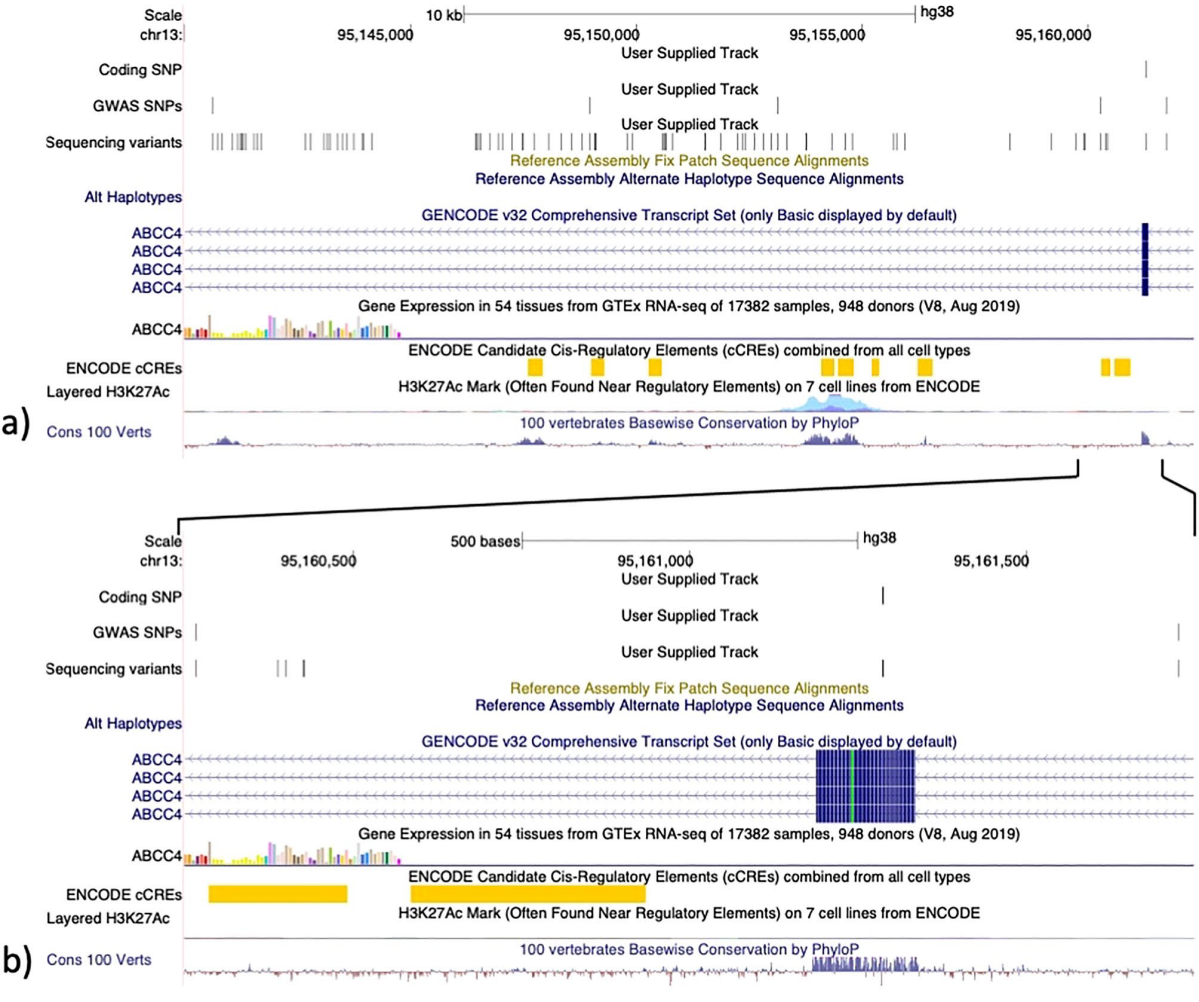
Detailed view of the associated locus on chromosome 22 lifted over to the human genome GRCh38. (**a**) Liftover of identified genetic variants located with the complete LD locus from CanFam3.1 to hg38. A separate track shows the location of the five associated GWAS SNPs and the identified coding variant within ABCC4. (**b**) Zoom in on the coding variant and the two nearest associated GWAS SNPs lifted over to human hg38. Tracks below the coding track include the ENCODE cis-regulatory elements track, the gene expression track, the Multix Alignments of 100 Vertebrates conservation track and the H3K27Ac regulatory elements track^9^.

In total 120 SNPs were identified within the ~ 18 kb haplotype block defined by the 5 SNPs in complete LD. Though the chr22:45,893,198 variant is the only coding variant within this region there are other variants in the locus located in genetically conserved regions or in cis-regulatory elements. In Fig. 4 we have visualized the SNPs lifted over to the human genome (hg38) in the UCSC browser^9^. In total, 85 of 120 SNPs could be lifted over to the human genome (hg38).

### Allele frequencies of the chr22:45,893,198 SNP in other breeds

Allele frequencies for the *ABCC4* candidate SNP chr22:45,893,198 were available in five other dog breeds and in a separate pool of American golden retrievers, from a Panel Of Normal (PON) dataset^10^. This dataset was collected as part of a cancer study unrelated to the current study and hence the dataset contained both male and female dogs and their disease history and neutering status is unknown. The allele frequency for the chr22:45,893,198 SNP in the population is summarized (Table S1). In this population of different dog breeds the allele frequency for the risk variant varies between from 0.37 in golden retrievers to 0.98 in Rottweilers. The Rottweiler is one of the breeds with the highest risk of developing pyometra (adjusted risk 4.4).

## Discussion

We performed a GWAS comparing golden retrievers affected by pyometra, with healthy intact female dogs older than 7 years of age. We found a genome-wide significant association to a region on chromosome 22 localized in the *ABCC4* gene. Sequencing data identified four non-synonymous SNPs in the *ABCC4 gene*. Genotyping of the coding SNPs in a larger cohort of golden retriever cases and controls showed that in particular one SNP, chr22:45,893,198, was in complete LD with the top-associated GWAS SNPs suggesting a potential causal function relating to the risk for development of pyometra in this breed, though other non-coding SNPs in this region could also be implicated. When performing a conditional analysis with the chromosome 22 associated SNP, a SNP on chr18:49,198,998 was shown to be more associated implicating that this complex disease is likely promoted by several genetic risk factors.

The ABCC4 protein, also known as multidrug resistant protein 4 (MRP4), is a member of the ATP-binding cassette transporter family, which encodes proteins that are important for transportation of endogenous and exogenous molecules across cell membranes^11,12^. Although ABCC4 has not previously been associated with uterine inflammation in dogs, it is known that the protein has central functions in the reproductive system, and its role in transporting prostaglandins (PGs) in the endometrium is well-established^11,12^.

Prostaglandins have many roles in reproduction^13,14^. The function and life-span of the *corpus luteum*, and physiology of parturition are regulated by complex interactions in which PGs participate^15^. PGs are also important inflammatory mediators and are produced and released from neutrophils, macrophages, lymphocytes, and platelets during inflammation. Importantly, uterine endometrial cells are capable of synthesizing and releasing PGs^16^. In dogs as well as many other mammals, circulating levels of PGs increase in uterine inflammatory conditions^16–22^. In the uterine tissue during canine pyometra, Prostaglandin-endoperoxide synthase 2 (*PTGS2*), a gene that is responsible for PG synthesis, is among the top genes for which expression is increased^23,24^. Furthermore, PGF_2α_ induces myometrial contractions, and is used therapeutically in medical treatment of pyometra in dogs^25,26^. Taken together, the many roles of PGs point to that altered prostaglandin transport could contribute to the development of pyometra^27,28^.

The associated coding sequence variant at chr22:45,893,198 causes an amino acid change, p.M787V. This amino acid is located in the cytoplasmic loop 3 (CL3) of the ABCC4 protein, a region of the protein that is phylogenetically conserved^29^. Across mammalian species the most common amino acid residue at the 787 position is Valine, and out of 61 mammals only the dog, pig, brush tailed rat, naked mole rat and chinchilla have Methionine in this position^9,30^. In this study the risk allele results in keeping the less common amino acid Methionine, whilst the protective allele results in a change into to the more common Valine. A recent paper described the importance of the cytoplasmic loop 3 and how a single amino acid substitution in ABCC4 p.T796M could reduce the expression and stability of the human ABCC4 protein. In that study the p.T796M ABCC4 substitution was predicted to be benign and well tolerated by SIFT and PolyPhen, however this was found to be unlikely by the authors due to the larger size of methionine^29^. In our study, the canine p.M787V ABCC4 substitution was also predicted to be benign and well tolerated by PolyPhen and SIFT. Nevertheless, it is possible that it could influence the ABCC4 cellular transportation capacity^29^.

The *ABCC4* risk variant is present in the canine reference genome sequence rather than the non-risk allele. The Canfam 3.1 assembly is based on a single individual (female boxer) and it is feasible that this individual carries the risk allele as the disease is common in boxers (diagnosed in 28% by 10 years of age, age adjusted relative risk 2.7)^2^. Though we predict that the coding sequence variant chr22:45,893,198 can influence the risk of developing pyometra, it is also possible that this SNP could increase the reproductive potential of the individuals, such as contributing to increased fertility, litter size, conception, or fetal growth and therefore, it could have been under selection. The susceptibility to disease is likely not selected against as the majority of pyometra cases develop after the main reproductive period.

The discrepancy between the association results in the smaller GWAS population and the larger TaqMan genotyping population can possibly be explained by the larger dataset including dogs which were excluded from the original GWAS dataset due to first degree relatedness. Hence the association analysis containing more dogs included many highly related individuals, which could falsely skew the data away from the original association. Due to the few variants in the dataset it is not possible to correct this association using a mixed model approach.

Interestingly, the chr22:45,893,198 SNP showed variation in allele frequency across breeds, with the Rottweiler being almost completely fixed for the risk allele. The Rottweiler is ranked as one of the dog breeds with the highest risk of developing pyometra (61% are diagnosed with the illness by 10 years of age)^1,2^. This suggests that this variant, could contribute to the disease risk in other breeds, however due to its high allele frequency it would be difficult to identify an association in the Rottweiler.

The ABCC4 transporter is known for its involvement in transporting drugs and other molecules across the cell membrane and altered function can lead to increased cellular toxicity in relation to exposure of various drugs. It is unknown whether the ABCC4 amino acid changes identified here can affect drug transport, but differences in NSAID transportation have been linked with altered ABCC4 function^31^.

In total 120 SNPs were identified in the associated GWAS locus. When lifted over to the human genome hg38, 85 SNPs could be transferred. Some of these SNPs were found to be located in areas with H3K27Ac histone enrichment and areas with a high conservation score based on data from Multiz alignments of 100 Vertebrates (Fig. 4)^9,30^. Even though this study focused on evaluating the coding mutations it is possible that other variants could have functional impact on gene expression or function and influence the risk for disease development.

Pyometra is likely induced by multifactorial genetic and environmental factors hence we investigated the potential of associated loci independent of the chr 22 locus and risk factors associated to age of onset. Though none of the other loci reached Bonferroni corrected significance, we detected suggestive associations to SNPs located within introns of several genes (*TESMIN*, *SORBS1*, *NAF1* and *EMCN*). Based on known function of the proteins encoded by these genes, potential implications for the development of pyometra could be considered^32–36^. A stronger association was seen to the SNP chr18:49,198,998, in the larger genotyping cohort. This SNP lies within the intronic region of *TESMIN,* The *TESMIN* gene encodes a metallothionein-like protein. In the mouse, this gene is expressed in both male and female reproductive organs and changes in expression have been observed in the endometrium in response to stress^37^. This locus was not studied in detail here though many additional potentially implicated genes were located within high LD of the associated SNP and additional genetic variants were identified in this region. Larger genome-wide association studies of more golden retrievers and other high-risk breeds will be necessary to achieve sufficient power to detect additional loci at genome-wide significance.

In conclusion, this GWAS identified an association to a locus in the *ABCC4* gene and subsequently identified a non-synonymous SNP in complete LD with the most highly associated GWAS SNP. Further validation studies are needed to establish the direct functional consequence of the *ABCC4* risk variant on the transport of prostaglandins and development of pyometra.

## Materials and methods

### Ethical approval

All methods were carried out in accordance with relevant guidelines and regulations. Samples were collected with the owners’ written informed consent and in agreement with Ethical guidelines. Ethical approval was granted by the regional animal ethics committee (Uppsala ethics committee on animal experiments/Uppsala djurförsöksetiska nämnd: Dnr C269/8, D318/9, C139/9, C41/12).

### Sample collection

Blood samples were collected from female golden retrievers affected by or with a history of pyometra. The dogs were identified through the diagnostic code for pyometra in the Agria Animal Insurance Inc. database, which has been validated for research purposes^38^. A questionnaire was filled in by each dog-owner directly prior to the time of blood sample collection. Details of the dog’s Swedish Kennel Club’s (SKK) registry number, name, age, birth date, previous whelping, onset of signs of pyometra, whether surgical treatment (ovariohysterectomy) had been performed or not, and past or present other diseases or medications were noted in the questionnaire. In parallel, blood samples were similarly collected from control dogs (> 7 years old intact female golden retrievers), identified via the SKK registry. Questionnaire and health information was also collected from the control dogs. Information regarding pedigree was extracted from the SKK database, based on the individual dog’s registration number. Siblings were excluded as to only include one individual in the case and control groups, respectively.

The health status of the control dogs was updated yearly by telephone contact, to assure that none of the controls had developed pyometra at the time of data analysis.

#### DNA extraction

Genomic DNA was extracted from EDTA blood by a robotic method using the QIASymphony robot (Qiagen, Hilden, Germany) together with the QIAamp DNA Blood Midi Kit (Qiagen).

#### Genome-wide genotyping

DNA from each dog was genotyped using the Illumina 170 K CanineHD BeadChip (Illumina, San Diego, CA, USA). A total of 98 cases and 96 control samples were genotyped on the NeoGen Genomics genotyping platform (NeoGen Genomics, Lincoln, NE, USA).

#### GWAS analysis

Data quality control and filtering was performed using the software PLINK 1.07^39^. SNPs were removed if they had a minor allele frequency (MAF) less than 5% or if they had failed to be genotyped in more than 5% of samples (–maf 0.05, –geno 0.05). Individuals with a genotyping rate of less than 95% were removed (–mind 0.05). To evaluate and visualize the population structure a multidimensional scaling (MDS) plot was generated on the filtered dataset using PLINK 1.07^39^. The first 4 coordinates (C1–C4) were calculated and the first two were plotted against each other to illustrate population structure. An additional control for relatedness within the cases or controls was calculated using PLINK v 1.9 by calculating PI_HAT on the final dataset after LD pruning^39^.

To account for cryptic relatedness within the population and population structure the GWAS analysis was performed using the efficient mixed model association expedited software (EMMAX)^8^. The software was used with an identity by state (IBS) matrix. QQ-plots and Manhattan plots were generated in R using the software package Lattice^40^. The significance threshold was determined using an LD corrected Bonferroni significance threshold based on 11,897 SNPs that were not in complete or near-complete LD as calculated by PLINK –indep 100 10 10 as previously described^41^.

A conditional association analysis to look for risk factors independent of the top associated loci was performed using the genotype for the associated SNP (chr22: 45,875,420) as a covariate in the GWAS analysis using EMMAX.

To search for risk factors associated with age of onset, age was calculated in days for all the cases. A GWAS analysis was then performed including only the cases and using the age of onset in days as a continuous variable using the EMMAX software.

### Whole genome sequencing

Whole genome sequencing data was generated from one pool of 10 golden retrievers, all heterozygous for the risk haplotype and from one dog homozygous for the risk haplotype. In addition, individually barcoded sequencing data was generated from 10 individuals homozygous for the non-risk haplotype (PRJNA693123). Samples were sequenced as paired-end libraries with 100 bp read length on the Illumina HiSeq2000 system. Data was aligned to the CanFam3.1 reference genome sequence (http://www.ncbi.nlm.nih.gov/assembly/317138) according to the Genome Analysis Tool Kit (GATK) best practices work flow^42^. Variants in the regions of interest were annotated using variant effect predictor^43^, including annotation with SIFT^44^ and PolyPhen-2^45^.

### TaqMan genotyping of SNPs in candidate region

TaqMan custom arrays (Table S2) were designed for genotyping of four non-synonymous coding SNPs in the *ABCC4* gene and using the Custom TaqMan Assay Design Tool (ThermoFisher Scientific, Waltham, MA, USA). Assays were also designed for two of the top GWAS SNPs, as controls and to evaluate genotypes for additional samples, which were not genotyped on the 170 k CanineHD BeadChip.

Genotyping was performed in 292 golden retrievers including 134 cases and 158 controls. This included 97 cases and 96 controls genotyped for the GWAS analysis and additional novel cases and controls. Unfortunately, one of the cases from the GWAS study could not be genotyped for the additional candidate SNPs due to lack of DNA. Manual imputation of the ABCC4 coding SNPs with an r^2^ above 0.99 to the original GWAS locus was performed for this individual. The basic association on the TaqMan genotyping data was carried out using PLINK 1.07^39^.

### Allele frequencies in panel of normal

Genotypes for the four coding SNPs in the *ABCC4* gene were extracted from a Panel of Normals (PON) database generated for a separate study which is displayed as a track on the UCSC genome browser^10^.

## Supplementary Information

Supplementary Information.
