# Supplementary material for: The ABCC4 gene is associated with pyometra in golden retriever dogs
Source: Sci Rep. 2021 Aug 17;11:16647. doi: 10.1038/s41598-021-95936-1 (PMC8370986; doi:10.1038/s41598-021-95936-1)
Supplement: Supplementary file 1 — Supplementary Information. [file 41598_2021_95936_MOESM1_ESM.docx]

**The *ABCC4* gene is associated with pyometra in golden retriever dogs.**

**Supplementary table S1, S2 , S3 and figure S1, S2, S3**

| **Breed** | **N** | **Adjusted risk pyometra** | **Risk Allele frequency 45893198-A** |
| --- | --- | --- | --- |
| Rottweiler | 24 | 4.4 | 0.98 |
| Boxer | 24 | 2.7 | 0.75 |
| Cocker spaniel | 15 | 2.8 | 0.83 |
| Labrador retriever | 24 | 1.8 | 0.77 |
| Standard poodle | 27 | Not reported | 0.91 |
| American Golden retriever | 115 | 3.3 | 0.37 |

**Table S1: The presence of the missense SNP in other breeds and American golden retrievers.** The allele frequency for the chr22:45893198 coding variant was evaluated from existing data. The allele frequency was compared to the adjusted risk as published by Egenvall et al. 2001^2^.

| **Primer name** | **Primer sequence** | **Reporter Name** | **Reporter 1 Dye** | **Reporter Sequence** | **Reporter Quencher** |
| --- | --- | --- | --- | --- | --- |
|  |  |  |  |  |  |
| Chr22_45893198_F | CATTTTGTTGTGCAAAGTTTGTGAAGAA | Chr22_45893198_V | VIC | AGATCTCTGTTGATGTTCTA | NFQ |
| Chr22_45893198_R | TTGACGGTGGCTACTGTTCTTTT | Chr22_45893198_M | FAM | ATCTCTGTTGGTGTTCTA | NFQ |
| Chr22_45934522_F | ACCGTACCTTCGCAAATTGGT | Chr22_45934522_V | VIC | TAAGATCCGCAAAGGAC | NFQ |
| Chr22_45934522_R | GCATAAGAATAATAAAAATGTATGCCTGGGAAA | Chr22_45934522_M | FAM | AAGATCCACAAAGGAC | NFQ |
| chr22_45815581_F | TTGTAATCATACCACAAATAGTTATTCACATCTGT | chr22_45815581_V | VIC | TGATTCTGGCAAAAAG | NFQ |
| chr22_45815581_R | GTTTGTCATTGTTGTTGCCTTTGG | chr22_45815581_M | FAM | CTGATTCTGACAAAAAG | NFQ |
| Chr22_45823359_F | GTTGCTAAGAAATATCGCCGAAGAA | Chr22_45823359_V | VIC | CCCTTGCCGTCATTT | NFQ |
| Chr22_45823359_R | TTGGATCGCAATACCTCTGCTT | Chr22_45823359_M | FAM | CCCTTGCCATCATTT | NFQ |
| Chr22_45882260_F | GCCAGCCTAACTAAATGTTACATTCC | Chr22_45882260_V | VIC | TGTGAAAGAAGATGTGAGCG | NFQ |
| Chr22_45882260_R | AGCTTTAATCCTTGATACCAAACATTCTGA | Chr22_45882260_M | FAM | TGTGAAAGAAGATATGAGCG | NFQ |
| Chr18_49198998_F | CAACAAGTGGATATGCCTAGAAGGA | Chr18_49198998_V | VIC | CCTTTCCCGAATTGG | NFQ |
| Chr18_49198998_R | ACACCACACCTGAACCATTAACC | Chr18_49198998_M | FAM | CCTTTCCCG**G**ATTGG | NFQ |

**Table S2: Primers and reporters used for the TaqMan genotyping.**

| **CHR** | **BP** | **Allele frequency affected** | **Allele frequency unaffected** | **P** | **OR** |
| --- | --- | --- | --- | --- | --- |
| 18 | 49198998 | 0.64 | 0.48 | 7.89E-05 | 0.5093 |
| 22 | 45815510 | 0.12 | 0.04 | 5.39E-04 | 3.107 |
| 22 | 45823359 | 0.0 | 0.003 | 3.60E-01 | 0.0 |
| 22 | 45882260 | 0.17 | 0.07 | 1.91E-04 | 2.67 |
| 22 | 45893198 | 0.18 | 0.07 | 1.48E-04 | 2.713 |
| 22 | 45934522 | 0.66 | 0.64 | 5.44E-01 | 0.8992 |

**Table S3**: Summary of the association analysis of the TagMan genotyped SNPs in the larger cohort of golden retrievers.

**Figure S1: Multidimensional scaling plot visualising genetic distance in cases and controls.**

Graph showing the two first dimensions C1 (x-axis) C2 (y-axis) as calculated by PLINK^38^. No major stratification is seen between cases and controls. Cases are indicated as black dots whilst controls are open circles.

a)

b)

c)

**Figure S2.** a) Diagram showing the genotype distribution of the associated SNPs on chr 22. b) Diagram showing the genotype distribution of the associated SNPs on chromosome 18. Red colour indicates presence of risk allele. c) Table showing the percentage of cases and controls carrying a risk allele.

**Supplementary figure S3.** Diagram showing the dogs included in the TaqMan genotyping dataset and further analysis.
